# Variational Mode Decomposition Weighted Multiscale Support Vector Regression for Spectral Determination of Rapeseed Oil and Rhizoma Alpiniae Offcinarum Adulterants

**DOI:** 10.3390/bios12080586

**Published:** 2022-08-01

**Authors:** Xihui Bian, Deyun Wu, Kui Zhang, Peng Liu, Huibing Shi, Xiaoyao Tan, Zhigang Wang

**Affiliations:** 1 State Key Laboratory of Separation Membranes and Membrane Processes, School of Chemical Engineering and Technology, Tiangong University, Tianjin 300387, China; wudeyun1221@163.com (D.W.); zhangkui@dicp.ac.cn (K.Z.); liupeng012@tiangong.edu.cn (P.L.); tanxiaoyao@tiangong.edu.cn (X.T.); wangzhigang@tiangong.edu.cn (Z.W.); 2 State Key Laboratory of Plateau Ecology and Agriculture, Qinghai University, Xining 810016, China; 3 Shandong Provincial Key Laboratory of Olefin Catalysis and Polymerization, Shandong Chambroad Holding Group Co., Ltd., Binzhou 256500, China; huibing.shi@chambroad.com

**Keywords:** variational mode decomposition, support vector regression, adulteration, quality control, chemometrics

## Abstract

The accurate prediction of the model is essential for food and herb analysis. In order to exploit the abundance of information embedded in the frequency and time domains, a weighted multiscale support vector regression (SVR) method based on variational mode decomposition (VMD), namely VMD-WMSVR, was proposed for the ultraviolet-visible (UV-Vis) spectral determination of rapeseed oil adulterants and near-infrared (NIR) spectral quantification of rhizoma alpiniae offcinarum adulterants. In this method, each spectrum is decomposed into *K* discrete mode components by VMD first. The mode matrix U*k* is recombined from the decomposed components, and then, the SVR is used to build sub-models between each U*k* and target value. The final prediction is obtained by integrating the predictions of the sub-models by weighted average. The performance of the proposed method was tested with two spectral datasets of adulterated vegetable oils and herbs. Compared with the results from partial least squares (PLS) and SVR, VMD-WMSVR shows potential in model accuracy.

## 1. Introduction

Quality control is a critical analytical topic, especially regarding foods and herbs that play an essential role in everyday life. Adulteration is one of the major challenges in the quality control of foods and herbs. Some unscrupulous vendors dilute them with cheap alternative food sources or fraudulently label and sell low-quality products as premium ones to make more profit [1]. These not only damage the quality and nutritional value of foods but also are detrimental to consumer health. With a series of food adulteration incidents per year that lead to severe health impacts and economic costs, the problem of food fraud has become more sinister and devastating in the globalized food supply chain [2,3,4]. An important index for evaluating the nutritional value and quality of foods and herbs is to determine the contents of the main components in them. However, it is difficult to determine adulterants in the final product as some foods or herbs are similar in appearance but vary greatly in price [5,6]. In order to protect consumer rights and ensure food safety, there is an urgent need to develop a simple and reliable method to meet the accurate quantitative analysis of food and herb adulteration.

Various techniques have been applied to detect adulteration in foods and herbs, such as chromatography, mass spectrometry and capillary electrophoresis. These methods allow for a relatively accurate determination of the samples [7,8]. However, most of them are time-consuming and expensive and require a high degree of technical expertise [9]. Spectroscopic techniques, especially ultraviolet-visible (UV-Vis) and near-infrared (NIR) spectroscopy, have been rapidly developed in scientific research and industrial production because of their non-contact, environmentally friendly and low-cost advantages [10,11,12,13]. Since the original spectra usually contain a large amount of signal overlap, background and noise information unrelated to the target, chemometric models need to be integrated to improve and expand the potential applications of the spectroscopic techniques.

Chemometrics, as an effective support means, has been developed extensively in analytical chemistry [14,15,16,17], especially in multivariate calibration methods for spectral data analysis, such as partial least squares (PLS) and support vector regression (SVR) [18,19]. PLS is a commonly used modeling method because of its practicality and versatility, but it may produce undesirable prediction results when dealing with strongly nonlinear issues [18]. SVR has the capability to solve both linear and nonlinear multivariate regression problems with a simple process [20]. These modeling approaches predict unknown samples by constructing one model, but the prediction performance of only a single model that is built between spectra and targets tends to be poor when the training set is small or the samples are outliers [21].

Ensemble modeling has gained increasing attention in the multivariate calibration for quantitative analysis [22,23]. Compared with the prediction of a single model, ensemble modeling achieves a greater accuracy and more robust results by combining the predictions of multiple sub-models to produce the final prediction [21]. One of its key points is the generation of training sub-sets that can be produced from samples, variables or both directions, such as bagging, cluster and boosting [24,25,26]. However, most spectra are essentially localized and have varying localization in time and frequency. These traditional ensemble strategies are all generated sub-models from the original data that do not use both time and frequency information of the signal simultaneously [27]. Due to the complexity of the spectra, if the original signal is decomposed by mathematical transformation before ensemble calibration, better results may be obtained. There is different information hidden in the data that can be revealed by converting signals from the original data space to other spaces through a certain mathematical transformation.

Three decomposition strategies are widely applied for the signal process, that is, the Fourier transform (FT) [28], wavelet transform (WT) [29] and empirical mode decomposition (EMD) [30]. FT portrays well the frequency domain information of signals, but it does not provide time domain information and can only deal with stationary and linear signals [31]. WT has displayed its modeling effectiveness owing to its capacity for time-frequency resolutions. Nevertheless, WT is not a self-adaptive decomposition that needs to choose wavelet filters and scales for a given application to obtain an optimal result [32]. EMD is a useful technique for processing non-stationary and nonlinear signals and decomposes the signal into a finite number of intrinsic mode functions (IMFs) [30]. Although this self-adaptive decomposition method is a potent tool for the multiscale analysis of data without the trouble of selecting the filters or scales, the existence of mode mixing and end effect in the EMD process will lead to the distortion of IMF components [33]. Therefore, it is necessary to develop a new mathematical transformation for the signal process, which can make up for the deficiency in the above methods.

Variational mode decomposition (VMD) is a new adaptive signal decomposition strategy that is particularly suitable for nonlinear and non-stationary signals [34]. It not only has a good separation effect on the noise in signals but also effectively suppresses the mode mixing and end effect [35]. Using VMD, a series of mode components can be decomposed from the complex spectra according to the inherent characteristics of the signals. Previously, various studies reported that VMD has been used successfully in multiple fields due to its efficiency superiority, such as the forecast of stock prices [36], wind speed forecasting [37] and fault diagnosis [38]. However, there are very few reports in the literature that use the VMD algorithm for ensemble modeling in the spectral determination of food. Since VMD can fully utilize the information embedded over the frequency and time domains of spectral signals, it was introduced in the generation of sub-models.

Herein, a weighted multiscale SVR modeling method based on VMD for improving the prediction accuracy of food and herb adulterants is proposed and referred to as VMD-WMSVR. Firstly, each spectral signal is decomposed by VMD and then *K* mode components with different central frequencies are obtained. After recombining these mode components, SVR is used to establish sub-models for each mode. Finally, the predictions of each sub-model are weighted and averaged to obtain the ultimate prediction result. The spectral datasets of adulterated vegetable oils and herbs were investigated using this method. The performance of the method was evaluated based on the root mean square error of prediction (RMSEP) and correlation coefficient (R) and compared with results derived from single PLS and SVR models.

## 2. Materials and Methods

### 2.1. Sample Preparation

For adulterated vegetable oils, the sample consists of six different vegetable oils bought in different markets in the municipality of Tianjin. These are sesame oil, soybean oil, corn oil, peanut oil, rapeseed oil and sunflower oil. The six pure oils were blended in different mass proportions in order to form 51 adulterated vegetable oil samples. Each oil content is within the range of 0–100% (g/g) with an interval of ca. 2%. Before measurement, these samples were well shaken and sonicated in an ultrasonic instrument (SK6200HP, Kudos Ultrasonic Instrument Company, Shanghai, China) for 30 min to further mix and eliminate air bubbles. In this study, rapeseed oil was taken as the analysis target.

For adulterated herbs, the sample includes pure herbs. These are *Panax notoginseng* (PN), rhizoma alpiniae offcinarum (RAO), rhizoma curcumae (RC) and *Curcuma longa* (CL) purchased from various pharmacies in Tianjin. Since the herbs have a certain amount of moisture, they were dried at 60 °C to a constant weight. These herbs were ground into powder, passed through a 120-mesh stainless steel sieve and stored in sealed plastic bags measuring 60 mm × 100 mm. The four processed herbs were mixed at different mass percentages and ensured that the total mass fraction of the four herbs in each sample was 100%. There were 75 samples in the adulterated herbs dataset and studied with the content of RAO.

### 2.2. Spectral Collection

**Figure 1:**
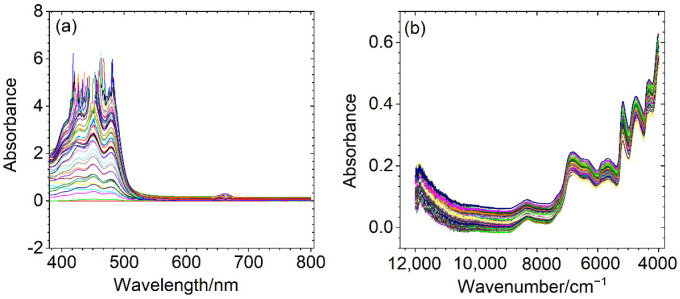
Measured spectra for adulterated vegetable oil (**a**) and herb (**b**) datasets.

Two small spectral datasets were experimentally investigated. A UV-Vis spectrophotometer (Evolution 300, Thermo Fisher, Waltham, MA, USA) was used for the adulterated vegetable oils in order to obtain the spectra of 51 samples in the wavelength range from 200 to 800 nm with an interval of 1 nm. The average spectrum of three parallel measurements was used for each sample. There is a negative absorbance for the 200–380 nm wavelengths, which seems to have no useful information. When the absorbance is above four, there is an obvious noise phenomenon and the absorption peaks are mainly present at 380–800 nm. Thus, Figure 1a mainly shows the absorption peaks at wavelengths of 380–800 nm. The adulterated herbs were measured from 12,000 to 4000 cm^−1^ at 2 cm^−1^ intervals on a Vertex 70 NIR spectrometer (Bruker Optics Inc., Ettlingen, Germany). Figure 1b shows the NIR spectra of the samples.

Each spectrum of the adulterated samples in the same dataset is similar. Therefore, it is necessary to combine multivariate calibration with spectroscopy to achieve an accurate quantitative analysis. Before calculation, the two datasets were divided into the training and prediction set by the Kennard–Stone (KS) algorithm. KS is the most widely used grouping method in chemometrics, which usually yields good grouping results. For the vegetable oil dataset, 34 and 17 samples are used as the training and prediction sets, respectively. For the herb dataset, 50 and 25 samples are used as the training and prediction sets, respectively.

### 2.3. Variational Mode Decomposition (VMD)

VMD is a powerful technique for signal analysis, which depends on the frequency information of the signal. The basic idea of the VMD algorithm is to construct and solve variational problems. For the construction of the variational problem, the purpose of VMD is to decompose the spectral signal **X** into a number of *K* discrete mode components $u_{k}\;$ around the center frequency $\omega_{k}$. At the same time, the sum of each mode is equal to the input signal **X**. The constrained variational model consists of the following target function.

(1) $$\{\begin{array}{c} \underset{\left\{u_{k},\;\;\omega_{k}\right\}}{\min}\left\{\underset{k}{\sum }||\partial_{t}\left[\left(\delta \left(t\right)+\frac{j}{\pi t}\right)*u_{k}\left(t\right)\right]e^{-j\omega_{k}t}|{|}_{2}^{2}\right\}\\ s.t.\underset{k}{\sum }u_{k}=X \end{array}$$

where $\left\{u_{k}\right\}=\left\{u_{1},\ldots ,u_{K}\right\}$ is the mode ensemble obtained by decomposition, $\left\{\omega_{k}\right\}=\left\{\omega_{1},\ldots ,\omega_{K}\right\}$ represents the center frequency of each mode component, δ  is the Dirac function, ${\left||\cdot |\right|}_{2}$ is the L2 distance, ∗ is the convolution, *j* is the imaginary unit and **X** is a [m×n] matrix containing *n* spectral responses of *m* samples.

By introducing Lagrange multipliers and quadratic penalty terms, the above problem can be transformed into an unconstrained variational problem. An alternate direction method of multipliers (ADMM) is used to solve the saddle points of the multipliers’ function.$\;\left\{u_{k}\right\}$, $\left\{\omega_{k}\right\}$ and the Lagrange multiplier are updated continuously in the frequency domain until the optimal solution of the variational problem is obtained. Finally, the results are derived by a FT. Please refer to Ref [34]. for the detailed algorithm.

### 2.4. Support Vector Regression (SVR)

SVR is a machine learning algorithm based on the principle of structural risk minimization and function approximation. It is specifically used to obtain predictive models via a number of identified support vectors and nonlinear kernel functions. The main process of SVR is to map the input data into a high-dimensional space by kernel functions. Then, the optimal hyper-plane is found in this feature space and a model is built to solve the linear regression problem. With strict statistical theory, SVR is able to be trained with few samples. Least square SVR (LSSVR) is one of the SVR algorithms. It can transform the quadratic programming problem into the problem of solving linear equations to reduce the complexity of computation. The Lagrangian function that is constructed to solve the linear system is as follows:

(2) $$\left[\begin{array}{c} 0\\ I_{n} \end{array}\begin{array}{c} I_{n}^{T}\\ K+\gamma^{-1}I \end{array}\right]\left[\begin{array}{c} b_{0}\\ b \end{array}\right]=\left[\begin{array}{c} 0\\ y \end{array}\right]$$

where $I_{n}\;$ is a [n×1] vector, **K** is a [n×1] kernel matrix, T is a transpose of a matrix or vector, ***γ*** is a weight vector, **b** is regression vector and *b*_0_ is the model offset.

In this study, the Gaussian radial basis function (RBF) kernel function was used:

(3) $$k_{i,j}=e^{{\frac{-\left|x_{i}-x_{j}\right|}{2\sigma^{2}}}^{2}}$$

where **x***_i_* and **x***_j_* denote the measured spectra of different samples and *σ* is the kernel width parameter. As we can see from Equations (2) and (3), the performance of the SVR model is mainly affected by two parameters, namely, *γ* and *σ*^2^. More details are provided in Refs [22,39].

### 2.5. Variational Mode Decomposition Weighted Multiscale Support Vector Regression (VMD-WMSVR)

**Figure 2:**
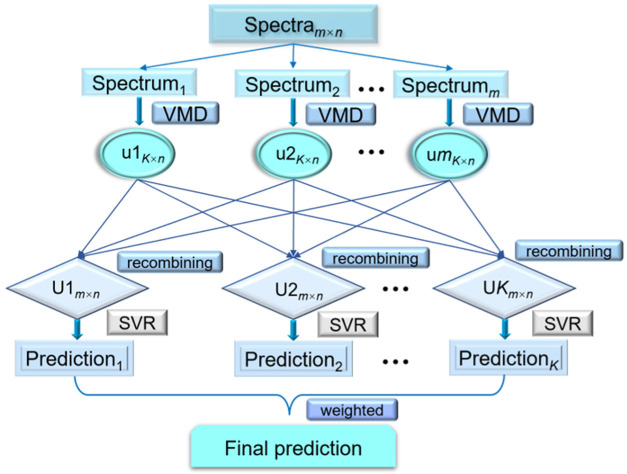
The schematic diagram of VMD-WMSVR.

Motived by the advantages of VMD and SVR, a novel ensemble modeling method (VMD-WMSVR) is proposed for the spectral quantitative analysis of food and herb adulterants. This method includes the calibration and prediction stages. The schematic diagram of the proposed method is shown in Figure 2. Details of the process are described as follows.

(1) Each spectrum of the training set is decomposed by VMD into *K* discrete mode components u*k* (*k* = 1, 2, …, *K*). Different *K* values of different samples have a large impact on the predictive stability of the proposed model. Moreover, too many mode components may destroy the linear relationship between the signal and the target value. The mode number *K* needs to be predetermined.

(2) Then, *K* mode components u*k* of the *i*th (*i* = 1, 2, …, *m*) spectral signal are assigned sequentially to the *i*th row of each corresponding mode matrix U*k*, i.e., the mode components u*k* are recombined to derive *K* modes U*k*. In this way, each U*k* contains the same number of samples and variables as the training set.

(3) SVR is used to build sub-models between each U*k* and the target values. Overall, *K* multiscale regression sub-models are established for calibration.

(4) In the process of prediction, the spectral decomposition and recombination of the prediction set is the same as that of the training set. VMD-WMSVR is used for predicting the samples in prediction set. Each sub-model gives a prediction and all the predictions are weighted and averaged to obtain the final prediction result. Sub-model weights are the inverse of the fourth power of the root mean square error of cross-validation (RMSECV).

## 3. Results and Discussion

### 3.1. The Mode Number K

**Figure 3:**
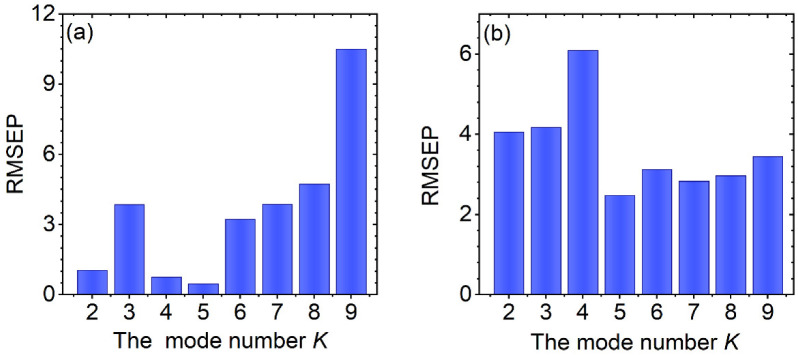
Variation in the RMSEP of VMD-WMSVR modeling with the mode number *K* for the adulterated vegetable oil (**a**) and herb (**b**) datasets.

In VMD-WMSVR model, the mode number *K* is a key parameter that needs to be set before the algorithm runs. Too few numbers of *K* may cause multiple components of the signal to be contained in one mode concurrently, resulting in insufficient decomposition. The model with too many numbers of *K* will create over decomposition problems and false modes [40]. In order to obtain the proper *K* value, the variation in the RMSEP with the mode number *K* for the two datasets is presented in Figure 3a,b, respectively.

VMD was applied multiple times with different *K* values for each spectrum and the result of the minimum RMSEP was considered the appropriate *K* value. Figure 3a shows that the RMSEP first presents a downward trend with the increase in *K*. When the *K* value reaches 5, the RMSEP obtains the minimum value and the prediction accuracy is the highest. After that, the RMSEP increases, especially when *K* is 9. This indicates that it may have mode mixing or pure noise modes that do not contribute much to the target value of interest. Figure 3b shows a similar trend to Figure 3a. As *K* is 5, the RMSEP reaches its minimum value. The smaller the RMSEP, the higher predictive accuracy of the model. Hence, the mode number *K* was set to 5 for both datasets.

### 3.2. The Spectral Decomposition of VMD

**Figure 4:**
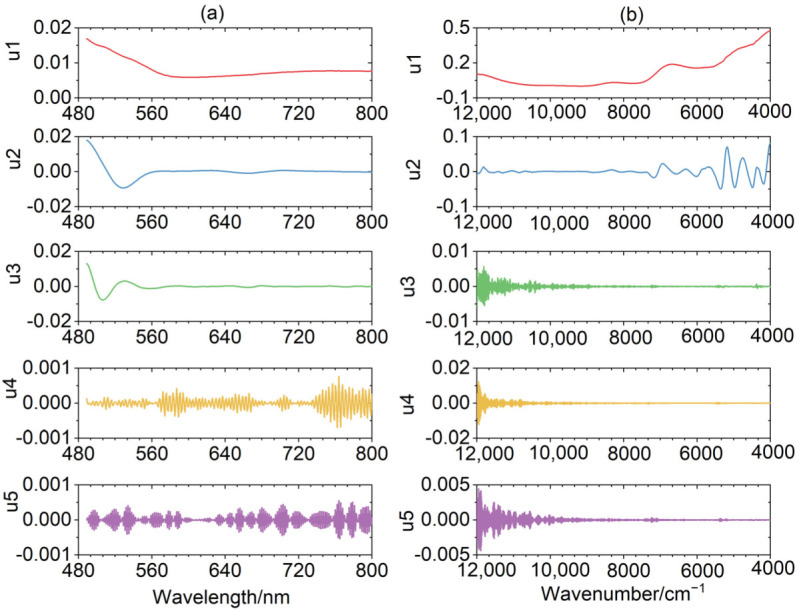
VMD diagram of the spectrum for sample No. 2 in the adulterated vegetable oil (**a**) and sample No. 17 in the adulterated herb (**b**) datasets.

With the determination of *K*, each spectrum of the training set is decomposed by VMD and obtains *K* discrete mode components u*k* using this method. The UV-Vis spectra of 34 samples for the adulterated vegetable oils in the training set are decomposed. To illustrate the decomposition result, sample No. 2 is used. Figure 4a demonstrates that the original UV-Vis spectra are decomposed into five u components, which are graphically explained in the extracted order. This order represents the change in frequency from the lowest frequency to the highest. Different frequency blocks may contain different information and contribute varyingly to the model. The first three u components fluctuate slightly with a low number of peaks and the wavelength fluctuation in the range of 560–800 nm is gentle, which may contain some useful information. For u4, it almost fluctuates symmetrically near zero over the entire wavelength range by detailed observation. In addition, big peaks alternate with small ones, making it difficult to determine whether they are noise or not. The variation in frequency for u5 is higher and the pronounced peak number increment is observed compared with the former u components, which behave more like noise.

For the adulterated herb dataset, each NIR spectrum of 50 samples in the training set is decomposed. Sample No. 17 is taken as an example. Figure 4b shows u1 oscillates slowly over the whole wavenumber with few peaks. Compared with u1, u2 changes more frequently and contains more peaks. Both of them have minor changes between 12,000 and 8000 cm^−1^ and have big fluctuations between 8000 and 4000 cm^−1^, which may include a lot of helpful information. The last three u components have similar trends throughout the wavenumber range, fluctuating almost symmetrically around zero. The variation in frequency is prominent, changing rapidly from 12,000 to 10,000 cm^−1^, which may have noise interference. In short, it can be seen from Figure 4 that the first u is a low-frequency component with a clear linear characteristic and a highly noticeable trend. As the order increases, the u component frequency becomes higher and higher, appearing with more irregularities and higher degrees of complexity. Since VMD is a mathematical decomposition, not all mode components have a well-defined chemical meaning for the spectral signal. The low-frequency and high-frequency mode components can be distinguished by observing their variation regularity combined with their variance values [41]. The low-frequency mode component changes gently with a large variance, while the high-frequency mode component oscillates almost symmetrically at zero with a small variance.

### 3.3. Comparison of the Predicted Results

**Figure 5:**
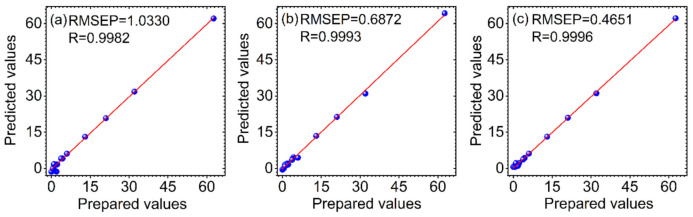
The relationship between the prepared and the predicted values for the prediction set by PLS (**a**), SVR (**b**) and VMD-WMSVR (**c**) for the adulterated vegetable oil dataset.

In order to evaluate the predictive ability of the proposed method, PLS and SVR are used for comparison. The parameters of PLS and SVR were optimized at first. For PLS, the Monte Carlo cross validation (MCCV) combined with the F-test was used to determine latent variables (LVs). The optimal LV for the adulterated vegetable oil and herb datasets is 5 and 4, respectively. For SVR, there are two parameters (*γ*, *σ*^2^) that need to be predetermined. The particle swarm optimization (PSO) algorithm was adopted and the RMSEP was used as the evaluation standard for the parameters optimization. The optimal *γ* and *σ*^2^ for the adulterated vegetable oil and herb datasets are 222.74, 227.37 and 247.46, 106.22, respectively. The relationship between the prepared and the predicted values for the prediction set by PLS (a), SVR (b) and VMD-WMSVR (c) for the two adulterated datasets is shown in Figure 5andFigure 6, respectively. The RMSEP and R of the prediction set are used as indicators to validate the performance of the models.

**Figure 6:**
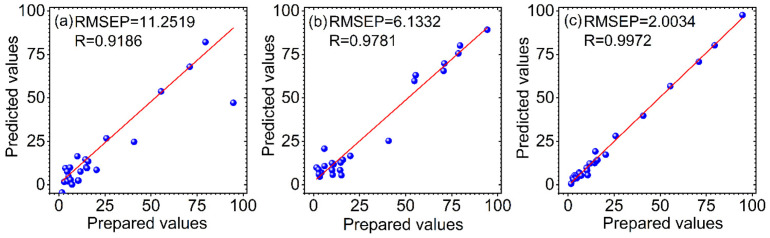
The relationship between the prepared and the predicted values for the prediction set by PLS (**a**), SVR (**b**) and VMD-WMSVR (**c**) for the adulterated herb dataset.

It can be found that the R values of the three methods are all above 0.9, indicating that these modeling methods combined with spectroscopy are effective for the quantitative analysis of rapeseed oil and RAO adulterants. However, the high benchmark of R leads to little room for improvement in all approaches, so their improvement is not significant from the perspective of R values alone. The variation in the RMSEP was used as the main criterion for comparison of the different methods. The RMSEP is a measure of the deviation between the predicted and prepared values. The smaller its value, the closer the predicted value is to the prepared value. For the vegetable oil dataset, it is observed from Figure 5 that SVR has a lower RMSEP and a higher R compared with PLS, demonstrating that SVR is superior to PLS. Among the three methods, VMD-WMSVR has the lowest RMSEP and the highest R. This indicates that the adaptive spectral decomposition can further improve the prediction ability of SVR and PLS. Thus, VMD-WMSVR has the best prediction, which is attributed to the original spectra of VMD. Figure 6 shows that compared with PLS, the RMSEP for the herb dataset under SVR is reduced by 45%. This also indicates that SVR is better than PLS in modeling results. There is a good linearity between the prepared values and the predicted values in Figure 6c. Compared with PLS and SVR, the RMSEP of VMD-WMSVR is reduced by 82% and 67%, respectively. Therefore, the prediction results of both datasets suggest the potential of VMD-WMSVR in improving the predictive accuracy.

## 4. Conclusions

In summary, this work presented a new chemometric methodology named VMD-WMSVR and was applied for two spectral datasets to achieve the quantification of vegetable oil and herb adulterants. On the one hand, VMD is designed to make full use of the information by decomposing the original spectra adaptively into multiple mode components with different frequencies. The modeling technique can improve the accuracy of predictions compared with a single PLS and SVR. On the other hand, it is a non-destructive and efficient method for the determination of rapeseed oil and RAO adulterants without the use of reagents and the generation of harmful residues, which protects the environment. However, the performance of predicting actual new samples was not discussed in this paper and should be further studied in the future.

## Data Availability

Not applicable.
